# Rhabdomyolysis as cause, consequence, or mimicker of myocardial infarction: A case report

**DOI:** 10.1002/ccr3.8133

**Published:** 2023-11-02

**Authors:** Marina Nasello, Mariachiara Ippolito, Antonino Federico, Fiammetta Ronga, Antonino Di Fede, Salvatore Campanella, Sara Accetta, Alessandra Gargano, Giulia Lo Scrudato, Lucrezia Urso, Antonino Giarratano, Andrea Cortegiani

**Affiliations:** ^1^ Department of Surgical, Oncological, and Oral Science (Di.Chir.On.S.) University of Palermo Palermo Italy; ^2^ Department of Anesthesia, Intensive Care, and Emergency Policlinico Paolo Giaccone University of Palermo Palermo Italy

**Keywords:** acute kidney injury, continuous renal replacement therapy, ICU, myocardial infarction, rhabdomyolysis

## Abstract

**Key Clinical Message:**

A timely diagnosis is essential to start appropriate therapy and to reduce risks of life‐threatening complications of rhabdomyolysis. Some cases can undergo differential diagnosis with other clinical conditions, e.g., myocardial infarction.

**Abstract:**

We present the case of a 65‐years‐old male who was admitted to the emergency department with a clinical presentation related to myocardial infarction. The patient underwent coronary angioplasty and was then admitted to ICU due to hemodynamical instability, elevated potassium levels, and anuria. Further investigations revealed rhabdomyolysis. The patient received vasopressors, oxygenation support and renal replacement therapy. Outcomes at ICU discharge were favorable. The temporal association between rhabdomyolysis and myocardial infarction, together with an unclear pathophysiological relationship, made differential diagnosis difficult. We discuss this uncertainty in light of published literature.

## 1 INTRODUCTION

Rhabdomyolysis is a medical condition characterized by an injury to skeletal muscles, causing the release of intracellular contents into the bloodstream, due to myolysis. Diagnostic criteria are the presence of elevated levels of cytolytic enzymes in the blood, such as myoglobin and creatine phosphokinase (CPK), with a value at least five times the upper limit of the normal range.^1^ Clinical presentation consists of weakness, fatigue, muscle pain, and dark urine due to myoglobinuria. Common causes of rhabdomyolysis are traumatic injuries, drugs (e.g., statins), abuse of alcohol, viral infections, long immobilization (i.e., long ICU stay), malignant hyperthermia, neuroleptic malignant syndrome, metabolic myopathies, heat strokes, endocrine, autoimmune, or rare genetic disorders.^2^ Potential consequences of an episode of rhabdomyolysis are electrolyte disturbances, disseminated intravascular coagulation (DIC), and acute kidney injury (AKI). The pathophysiological mechanism of AKI consists of renal vasoconstriction, myoglobinuric casts in the tubular lumen, and myoglobin‐direct cytotoxic effects on tubular cells induced by hemeprotein and free radicals.^3^

The kinetics of cytolytic enzymes is peculiar in rhabdomyolysis. Usually, myoglobin increases rapidly, reaching a plateau within 24 h and then it is eliminated, as fast as renal function is preserved. CPK has a longer rising time, as it increases in 2–12 h, with a peak in 3–5 days after muscle damage, and then it has a slower decrease in around 6–10 days.^4^

Like rhabdomyolysis, myocardial infarction (MI) is characterized by the release of intracellular contents, it is diagnosed with clinical presentation, EKG, and elevated myocardial biomarkers, released in the bloodstream after cardiomyocyte damage, such as troponin.

The reduction of blood flow through the coronary artery causes cellular death and cytolysis with subsequent release of intracellular contents. Troponin levels increase within 2–3 h from chest pain, with a peak in 12–24 h.^5^ An increase in CPK and myoglobin can also be seen in myocardial infarction, with kinetics similar to those caused by rhabdomyolysis. Both in rhabdomyolysis and myocardial infarction myoglobin increases early, however it's not considered a specific marker of MI. The clinical presentation also shares some common symptoms and signs, such as weakness and fatigue. MI symptoms include pain to chest, arm, abdomen or jaw, shortness of breath, nausea and vomiting. In rhabdomyolysis pain is usually widespread to skeletal muscles.

We present the case of a 65‐year‐old male who was admitted to the emergency department with symptoms related to MI, and later on, diagnosed with rhabdomyolysis, with an unclear pathophysiological relationship and differential diagnosis. The case was reported according to the CARE guidelines on reporting.^6^

## 2 CASE PRESENTATION

A 65‐year‐old male was admitted to the emergency department due to fatigue, myalgia, and altered mental status. Past medical history included chronic ischemic heart disease, hypertension, diabetes, chronic obstructive pulmonary disease, and mild chronic kidney disease. He had been surgically treated with CABG in 2022. At admission, he was diagnosed with acute MI, basing on clinical presentation, EKG and myocardial biomarkers. EKG showed atrial fibrillation, left bundle branch block, ST depression, and high and pointed T waves. Myocardial biomarkers were increased, T troponin was 532 ng/L (normal value <14 ng/L) and NT‐proBNP was 5579 ng/L (normal value <125 ng/L). A temporary pacemaker was positioned, for the onset of a bradyarrhythmia. Coronary angiography revealed: moderate calcified parietal atheromasia in common trunk, a critical stenosis of about 80% in anterior interventricular artery, diffusely atheromatous and distal stenosis in circumflex artery, diffuse parietal atheroma in right coronary artery with both a proximal and distal sub‐occlusive stenosis, occlusion of the right posterolateral branch venous bypass, patent, and well‐functioning arterial bypass for the anterior descending artery. In conclusion a severe three‐vessel coronary atheromasia was evidenced, compatible with the patient's chronic ischemic cardiac disease. Coronary angioplasty with drug‐eluting stent placement in the right coronary artery was performed with success. During the procedure a severe state of cardiogenic shock occurred, treated with vasopressor and inotrope drugs. Moreover, arterial blood gas test revealed a severe metabolic acidosis, along with an increasingly severe hyperkaliemia (7.5 mmol/L), and hemodynamic instability. The patient was consequently admitted to the Intensive Care Unit (ICU). Further laboratory tests were then performed, showing a CPK >22,000 U/L (normal range 39–308 U/L), myoglobin >30,000 μg/L (normal range < 72 μg/L), AST 1406 U/L (normal range < 50 U/L), LDH 1969 U/L (normal range 50–250 U/L), urea 304 mg/dL (normal range 10–71 mg/dL) and serum creatinine 6.58 mg/dL (normal range 0.67–1.17 mg/dL). Moreover, the patient complained severe legs and shoulders pain and a worsening inability to walk independently over the days preceding the ED admission. A diagnosis of rhabdomyolysis and AKI was thus posed. The patient was awake and cooperative, with a Glasgow Coma Scale of 15, in spontaneous breathing with oxygen supplement through a Venturi mask, but his hemodynamic was supported by continuous infusion of norepinephrine at high doses. Invasive blood pressure achieved 90/60 mmHg, lactate value reached 1.1 mmol/L with 0.5 mcg/kg/min of norepinephrine and fluid optimization, EKG showed regular activation from pacemaker Ventricular Ventricular Inhibiting mode 70 beats per minute and he was anuric.

Early Continuous Renal Replacement Therapy (CRRT) with adsorbent filter (i.e., CytoSorb®) was started for CPK and myoglobin removal and infusion of bicarbonates, diuretics, and mannitol was also initiated as supportive measures to preserve native renal function. Double antiplatelet therapy with aspirin and clopidogrel was started. During the ICU stay, cytolytic enzymes decreased with a gradual trend (see Table 1). Additional treatment consisted of oxygen administration through High Flow Nasal Cannula at 50 L/m flow and 0.5 FiO2, initiated at day 2 for the management of mild acute hypoxemic respiratory failure and to limit spontaneous respiratory drive, that had led to mild hypocapnia. The patient also received a transfusion with concentrated red blood cells. During the ICU there was a gradual but constant improvement of hemodynamics, clinical status, lab tests, and residual urine output, and the patient was transferred to the Coronary ICU at day 5. At 24 days, he was discharged from the hospital alive and with no residual worsening of kidney injury.

**TABLE 1:** Arterial blood gas and lab results.

	Day 0	Day 1	Day 2	Day 3	Day 4
CPK (U/L)	>22,000	74,192	19,521	6626	3395
Myoglobin (μg/L)	NA	>30,000	8777	4522	1428
Potassium (mmol/L)	7.3	5.8	4.4	3.9	3
Creatinine (mg/dL)	6.83	5.14	3.53	2.46	1.89
AST (U/L)	1406	1354	695	424	274
LDH (U/L)	1969	2147	1039	686	513
pH	7.26	7.34	7.42	7.44	7.44
PO2 (mmHg)	92	84	170	161	117
PCO2 (mmHg)	33	42	32	36	37
HCO3‐ (mmol/L)	14.8	22.7	20.8	24.5	25.1
FiO2 (%)	50	50	40	50	50
P/F Ratio (mmHg)	184	168	425	322	234
Respiratory Support	Venturi Mask	Venturi Mask	HFNC at 50 L/min	HFNC at 50 L/min	HFNC at 50 L/min

## 3 DISCUSSION

We presented a case of differential diagnosis between myocardial infarction and rhabdomyolysis in a patient with a past medical history of diabetes mellitus and chronic kidney disease. The patient had a clinical and laboratory presentation suggestive for MI, but further lab tests revealed increased cytolytic enzymes and a diagnosis of rhabdomyolysis was posed. According to guidelines, from the ED the patient was first treated by cardiologists with an urgent coronarography and the positioning of a stent in right coronary artery, because EKG and T troponin were suggestive of myocardial infarction. Despite both diabetes mellitus and chronic renal failure can lead to rhabdomyolysis, this diagnosis was not considered at the ED. The patient was then admitted to the ICU for persistent life‐threatening clinical condition, where the diagnosis of rhabdomyolysis become cleared and then confirmed. The patient was treated with fluids, early CRRT using Cytosorb® adsorbent filter, mannitol, and bicarbonates. There is no strong clinical evidence regarding the use of mannitol and bicarbonates, although their use can lead to renal vasodilatation and clearance of free radicals, with potential for renal function preservation, their use in patients with rhabdomyolysis is still controversial.^1^ Early CRRT is associated with a reduction of mortality, as it preserves renal function, thanks to the removal of myoglobin and CPK, and reduction of their deposition on renal tubules.^7^ The addition of adsorbent filters to the circuit contributes to effectively reduce myoglobin and CPK levels and to improve patient outcome.^8^, ^9^ CytoSorb® is a sorbent filter characterized by a biocompatible polymer with high porosity and a surface capable of absorbing hydrophobic substances with medium‐low molecular weight, it is indicated to remove cytokines, bilirubin, and myoglobin. Main indications are septic shock, rhabdomyolysis, remotion of anticoagulant molecules such as apixaban, rivaroxaban and ticagrelor in cardiac surgery.^10^

After 72 h of CRRT, the patient showed an improvement of renal function with gradual reduction of solutes, improvement of serum creatinine and resolution of electrolyte disorders, not previously responsive to medical treatment.

The complexity of this case was due to a differential diagnosis between cardiogenic shock subsequent to myocardial infarction and rhabdomyolysis determining shock. Other literature cases presented the same difficulty in distinguishing medical conditions with similar presentation. The cause primarily triggering this clinical presentation remained unclear, as common triggers of rhabdomyolysis (e.g., statins) were ruled out. Association between MI and rhabdomyolysis has been reported, usually occurring after cardiac arrest, CPR, and defibrillation^11^, ^12^; and defibrillation was identified as the causative agent of a muscular damage. Alternatively, electrolytic alterations induced by a rhabdomyolysis of unknown cause may mimic a MI in a patient with history of chronic ischemic heart disease.^13^

We can speculate that this was the pathophysiological course of our case. Indeed, potassium disorders can cause EKG alterations including peaked T waves and bradyarrhythmia, present in our patient's EKG. According to another possible explanation, a single causative factor can have affected myocytes and skeletal muscle cells simultaneously,^14^ but it wasn't possible to identify a common causative agent in this specific case.

## 4 CONCLUSIONS

Rhabdomyolysis can mimic or present along with myocardial infarction. The pathophysiological relationship between MI and rhabdomyolysis remained unclear in this case, but evaluation of CPK and myoglobin was useful to diagnose and to rapidly treat rhabdomyolysis, avoiding DIC and irreversible renal damage.

A timely diagnosis is essential to start early appropriate therapy and to reduce risks of life‐threatening complications of rhabdomyolisis.

## AUTHOR CONTRIBUTIONS

**Marina Nasello:** Conceptualization; data curation; validation; writing – original draft; writing – review and editing. **Mariachiara Ippolito:** Conceptualization; data curation; validation; writing – original draft; writing – review and editing. **Antonino Federico:** Data curation; validation; writing – review and editing. **Fiammetta Ronga:** Data curation; validation; writing – review and editing. **Antonino Di Fede:** Data curation; validation; writing – review and editing. **Salvatore Campanella:** Data curation; validation; writing – review and editing. **Sara Accetta:** Data curation; validation; writing – review and editing. **Alessandra Gargano:** Data curation; validation; writing – review and editing. **Giulia Lo Scrudato:** Data curation; validation; writing – review and editing. **Lucrezia Urso:** Data curation; validation; writing – review and editing. **Antonino Giarratano:** Data curation; validation; writing – review and editing. **Andrea Cortegiani:** Conceptualization; data curation; validation; writing – original draft; writing – review and editing.

## FUNDING INFORMATION

None.

## CONFLICT OF INTEREST STATEMENT

Mariachiara Ippolito is an Associate Editor for Clinical Case Reports. All the other authors declare no conflicts of interest.

## CONSENT

The patient provided written informed consent for publication of this case report.

## Data Availability

Data are available upon reasonable request to the corresponding author.
